# Low Bone Strength Is a Manifestation of Phenylketonuria in Mice and Is Attenuated by a Glycomacropeptide Diet

**DOI:** 10.1371/journal.pone.0045165

**Published:** 2012-09-18

**Authors:** Patrick Solverson, Sangita G. Murali, Suzanne J. Litscher, Robert D. Blank, Denise M. Ney

**Affiliations:** 1 Department of Nutritional Sciences, University of Wisconsin, Madison, Wisconsin, United States of America; 2 Geriatrics Research, Education, and Clinical Center, William S. Middleton Veterans Hospital, Madison, Wisconsin, United States of America; 3 Division of Endocrinology, Diabetes and Metabolism, Department of Medicine, University of Wisconsin, School of Medicine and Public Health, Madison, Wisconsin, United States of America; University of Munich, Germany

## Abstract

**Purpose:**

Phenylketonuria (PKU), caused by phenylalanine (phe) hydroxylase loss of function mutations, requires a low-phe diet plus amino acid (AA) formula to prevent cognitive impairment. Glycomacropeptide (GMP), a low-phe whey protein, provides a palatable alternative to AA formula. Skeletal fragility is a poorly understood chronic complication of PKU. We sought to characterize the impact of the PKU genotype and dietary protein source on bone biomechanics.

**Procedures:**

Wild type (WT; *Pah^+/+^*) and PKU (*Pah^enu2/enu2^*) mice on a C57BL/6J background were fed high-phe casein, low-phe AA, and low-phe GMP diets between 3 to 23 weeks of age. Following euthanasia, femur biomechanics were assessed by 3-point bending and femoral diaphyseal structure was determined. Femoral *ex vivo* bone mineral density (BMD) was assessed by dual-enengy x-ray absorptiometry. Whole bone parameters were used in prinicipal component analysis. Data were analyzed by 3-way ANCOVA with genotype, sex, and diet as the main factors.

**Findings:**

Regardless of diet and sex, PKU femora were more brittle, as manifested by lower post-yield displacement, weaker, as manifested by lower energy and yield and maximal loads, and showed reduced BMD compared with WT femora. Four principal components accounted for 87% of the variance and all differed significantly by genotype. Regardless of genotype and sex, the AA diet reduced femoral cross-sectional area and consequent maximal load compared with the GMP diet.

**Conclusions:**

Skeletal fragility, as reflected in brittle and weak femora, is an inherent feature of PKU. This PKU bone phenotype is attenuated by a GMP diet compared with an AA diet.

## Introduction

Phenylketonuria (PKU; OMIM 261600) is a recessive genetic disease of amino acid (AA) metabolism caused by loss of function mutations of the gene encoding phenylalanine hydroxylase (EC 1.14.16.1, *PAH* in humans and *Pah* in mice), resulting in an inability to convert phenylalanine (phe) to tyrosine [1]. PKU results in gross elevations of phe concentrations in tissue and blood, with downstream cytotoxicity, culminating in profound cognitive impairment if left untreated. Fortunately, this can be averted with lifelong adherence to a low-phe diet that excludes all high protein foods and requires an AA formula to meet nutrient needs [2]. With implementation of newborn screening for PKU in 1960–1970, there are an estimated 50,000 individuals worldwide with treated PKU and a normal range of cognitive function. Skeletal fragility in early adulthood has emerged as a chronic complication of PKU treated with a low-phe AA diet [3], [4], [5], [6], [7], [8], [9], [10], [11]. Because a low-phe AA diet is the standard of care and is instituted shortly after birth, it remains unknown whether bone fragility in PKU is inherent to the PKU genotype or secondary to its essential dietary management [3].

Compliance with the low-phe diet is often poor after early childhood owing to limited food choices and the bitter taste and strong odor of AA formulas [12], [13], [14], [15]. Moreover, a number of suboptimal outcomes in patients with PKU treated with diet have been identified [16]. Glycomacropeptide (GMP), a whey protein produced during cheese making, provides an alternative to AA formula because pure GMP contains no phe and can be made into a variety of low-phe, high protein foods and beverages for those with PKU [17]. Studies in humans with PKU indicate that GMP improves protein retention, phe concentrations, and palatability of the low-phe diet compared with AA formula [18], [19], [20]. Long term studies in the PKU mouse model (*Pah^enu2^*) demonstrate that a GMP diet supports similar growth and accretion of lean body mass and attenuates indices of metabolic stress compared with an AA diet [21], [22]. The evidence suggests that the GMP diet provides an acceptable, physiologic source of low-phe dietary protein that may also impact bone development for PKU.

In order to distinguish the contributions of the PKU genotype itself and dietary treatment of the disease, we have conducted a factorial experiment in PKU (*Pah* ^enu2/enu2^ *)* and wild type (WT, *Pah* ^+/+^ *)* mice fed casein, AA and GMP diets. The objective was to characterize the impact of the PKU genotype and dietary protein source on bone biomechanical performance. We assessed the femora by 3 point bending, allowing us to obtain information regarding bone strength (load and stress) and brittleness (displacement and strain), distinct mechanical properties that both contribute to fracture susceptibility [23]. Measuring the bones after testing allowed us to assess the contribution of cross-sectional bone geometry to mechanical performance. This is the first report to rigorously establish the separate contributions of genotype and diet to skeletal fragility in PKU.

## Materials and Methods

### Animals and Experimental Design

**Figure 1:**
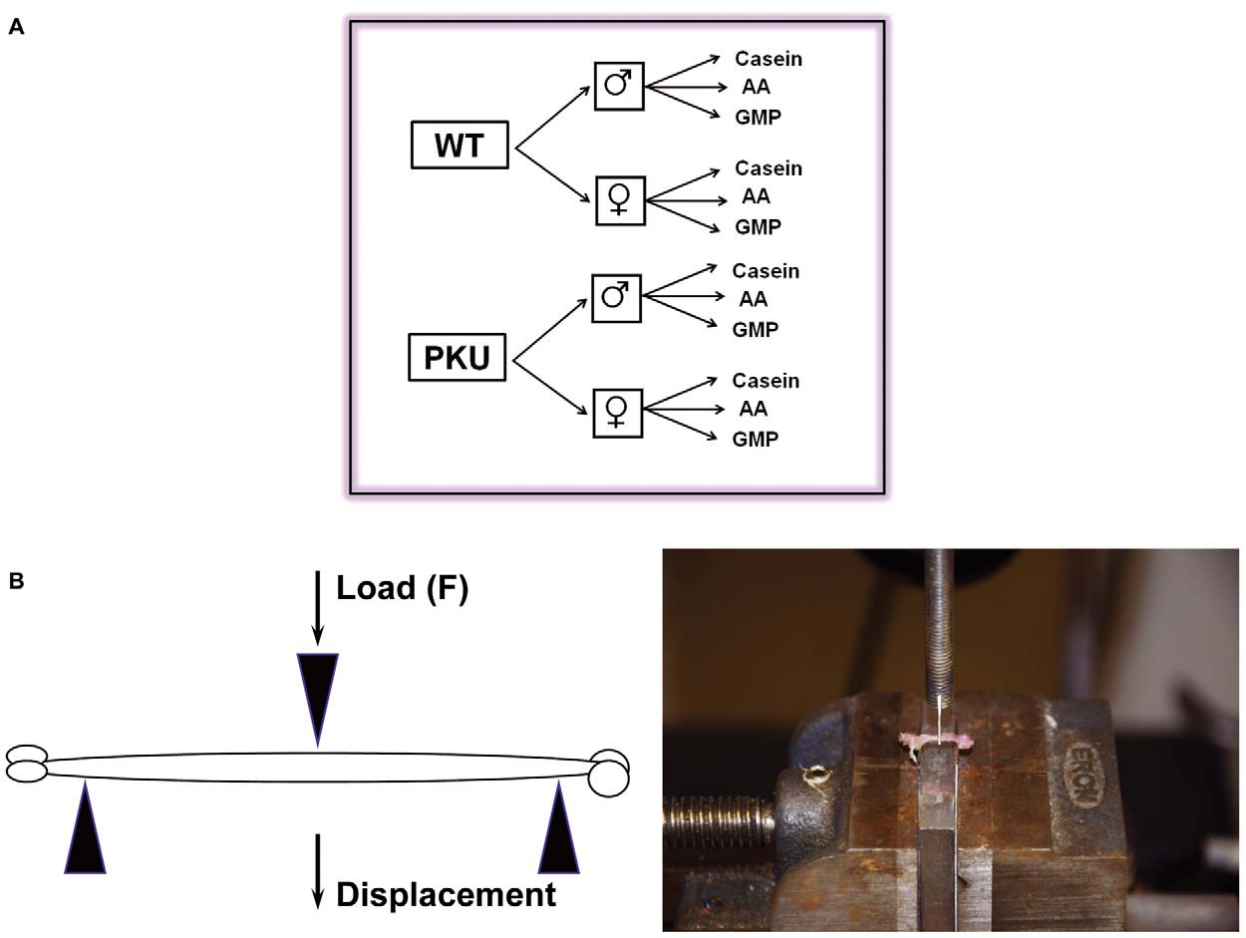
Experimental design and three-point bending test. The experiment utilized a 2×2×3 factorial design with a total of 12 groups (A). A cartoon of the three-point bending test of a mouse femur and a representative photograph (B).

The University of Wisconsin-Madison Institutional Animal Care and Use Committee approved the facilities and protocols used in this study. A breeding colony of PKU mice was used to produce experimental animals by breeding C57BL/6J mice heterozygous for the *Pah* ^enu2^ mutation to yield homozygous PKU mice and WT control mice [21], [24]. Experimental mice were genotyped for the presence of the *Pah* ^enu2^ mutation as described previously [22]. The experiment controlled for three main effects and their interactions in a 2×2×3 factorial design: genotype (WT or PKU), sex (male or female), and diet (low-phe GMP, low-phe AA, or high-phe casein) with casein serving as a control diet, **Figure 1A**. Mice were randomized to diet, separated by sex and housed within their litters at the time of weaning (21d) in shoe-box cages. Mice had free access to food and water and the facility was maintained at 22°C on a 12∶12-h light-dark cycle.

Mice were fed the experimental diets from weaning through young adulthood (3–25 weeks of age), which resulted in mice being fed diet for 20.4±0.11 weeks on average (range 17–22 weeks of feeding, n = 217 mice). The casein, AA and GMP diets were isoenergetic and the source of protein was the only variable manipulated (Harlan Teklad, Madison, WI; TD.09667 - TD.09669), as previously reported [22]. The casein diet included 20% (wt/wt) casein plus 0.3% L-cystine, the AA diet included 17.5% free AAs [25] and the GMP diet included 20% GMP (BioPURE GMP, Davisco Foods International, LeSueur, MN) plus 1.5 times the NRC requirement (equivalent to a total supplementation of 2.8% AA) for 5 limiting AA in order to provide a complete source of protein. The AA profile of the diets was previously reported [22]. All three diets were supplemented with approximately 10% more than NRC requirements for calcium, phosphorus and magnesium to optimize bone growth.

**Table 1:** In vivo measurements.

	Wild Type Mice						PKU Mice					
	Males			Females			Males			Females		
Variable	Casein	AA	GMP	Casein	AA	GMP	Casein	AA	GMP	Casein	AA	GMP
*N*	17 (10)	18 (7)	19 (7)	23 (9)	16 (4)	22 (10)	18 (7)	16 (5)	14 (6)	19 (6)	20 (4)	13 (3)
Body Mass (g)a,b,c,e,g	31.1±0.8	28.5±0.5	28.1±0.8	22.5±0.3	22.5±0.3	21.9±0.4	26.2±0.4	29.5±1.0	26.8±0.7	21.4±0.4	22.0±0.3	21.5±0.5
rPlasma phe (µmol/L)a,c,e	51.5±4.5	38.5±7.0	41.0±9.0	50.2±1.6	34.3±6.1	41.9±2.2	2026±151	726±61	745±26	2206±76	719±19	786±24
BMC (mg)a,c,d,f	574±13	533±12	528±13	533±18	527±15	602±14	571±16	504±14	555±15	489±16	495±11	542±8
BMD (mg/cm^2^)a,b,f	51.4±0.3	50.7±0.4	50.2±0.6	51.6±0.6	51.4±0.6	52.2±0.4	49.8±0.3	50.1±0.7	48.2±0.6	48.5±0.5	50.4±0.3	49.9±0.6
Length (mm)a,b,e	16.91±0.01	16.86±0.02	16.91±0.01	16.89±0.02	16.84±0.02	16.88±0.01	16.86±0.01	16.84±0.03	16.85±0.01	16.83±0.01	16.87±0.02	16.81±0.01

Values are means ± SE; *N*, no of mice for measurement of body mass; BMC, whole-body bone mineral content; BMD, whole-body bone mineral density.

r Data analyzed by 3 way ANOVA on ranked data.

a genotype effect,

b sex effect,

c diet effect,

d gt*sex effect,

e gt*diet effect,

f sex*diet effect,

g gt*sex*diet effect.

**Body mass:** WT female mice weighed more than PKU female mice. WT males fed casein and PKU males fed AA weighed the most. There was no difference in body mass between WT males fed AA and WT or PKU males fed GMP, and PKU males fed casein weighed the least.

**Plasma phe:** PKU mice had higher plasma phe than WT mice. Among PKU mice, there was a significant reduction in plasma phe with the GMP or AA diet. Among WT mice, there was also a significant reduction in plasma phe with the GMP or AA diet.

**BMC:** Females fed GMP and males fed casein had the highest BMC. There was no difference between females fed casein and males or females fed AA, but males fed GMP had a higher BMC than females fed AA. There was a significant reduction in BMC in PKU females compared to either WT females or WT and PKU males.

**BMD:** PKU animals had a lower BMD compared to WT animals. Males fed GMP had a lower BMD compared to females fed GMP or AA and males fed casein. Females fed casein had a lower BMD than females fed GMP.

**Length:** Males had longer femurs than female mice. WT mice fed AA had a significant reduction in femur length compared to WT mice fed GMP and casein. Moreover, PKU mice fed casein, AA and GMP had significantly shorter femurs than WT mice fed GMP and casein, but were not different from WT mice fed AA. Numbers in parenthesis indicate sample size from femur length analysis.

Dual-energy x-ray absorptiometry (DXA) with PIXImus software version 2.10 (GE/Lunar Corp, Madison, WI) was performed to obtain in vivo whole body bone mineral density (BMD) and bone mineral content (BMC) at the end of the experiment. Mice were anesthetized with isoflurane with an anesthesia machine (IsoFlo, Abbott Laboratories, North Chicago, IL) and placed prone on the DXA scanner bed with their tail and appendages fully extended. Each mouse received one scan at the completion of the study. Handling of the data obtained from the DXA scan was performed by a single scientist blinded to the treatment groups and subsequent statistical analysis of the densitometry data was performed by the authors. Once mice completed the feeding study, they were placed under anesthesia using an isoflurane anesthesia machine and euthanized by exsanguination via cardiac puncture. Following euthanasia, both femora were dissected free of soft tissue, wrapped in phosphate buffered saline-saturated gauze, and stored at −80°C. Specimens were subjected to two freeze thaw cycles, one prior to DXA, and the second prior to biomechanical testing. Using DXA we measured areal BMD of isolated femura twice with repositioning, as previously described [26]. Prior to biomechanical analysis, femora were gradually warmed by placing them at 4°C for at least 12 hours, and then allowing them to come to room temperature prior to analysis. Bones were broken in two different sessions. Prior to bone fracture femur length of the second block of femora (n = 78 mice) was measured using Vernier calipers measuring the distance between the greater trochanter and the medial condyle.

**Figure 2:**
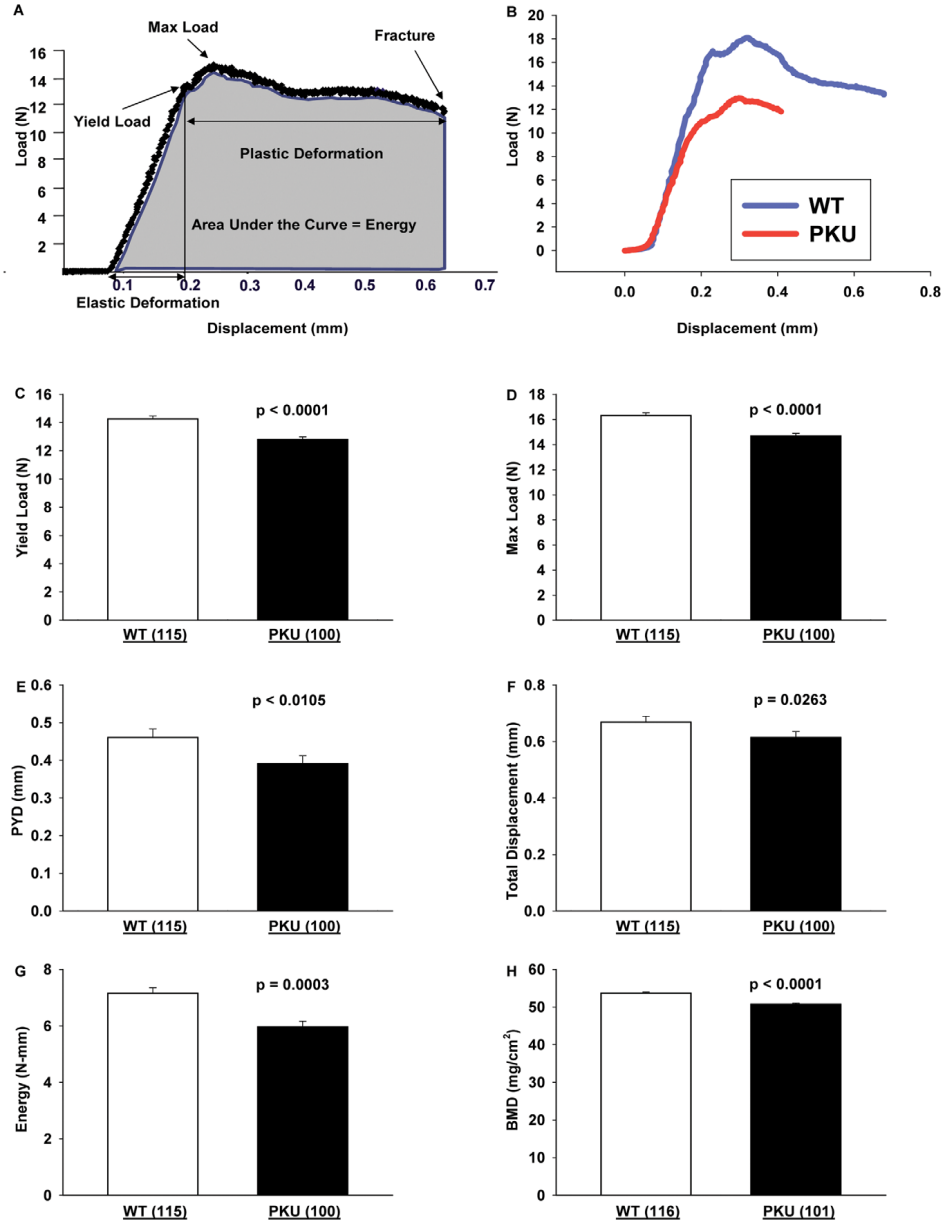
Force-displacement curve analysis of WT and PKU mice. Schematic of a load-displacement curve generated from the three-point bending test from which the yield point, maximum load, elastic and plastic deformation, and energy to failure (shaded area under the curve) are obtained (A). Representative load-displacement curves for WT and PKU mice (B). Effects in WT and PKU mice for yield load (C), maximum load (D), post-yield displacement (PYD) (E), total displacement (F), energy to failure (G), and femoral bone mineral density (BMD) (H). Values are means ± SE; p-values represent main effect of genotype. Sample size is shown in parenthesis. All values for femoral biomechanical performance had a significant main effect for genotype, WT >PKU.

**Table 2:** Force-displacement curve analysis showing whole bone biomechanical performance.

	Wild Type Mice						PKU Mice					
	Males			Females			Males			Females		
Variable	Casein	AA	GMP	Casein	AA	GMP	Casein	AA	GMP	Casein	AA	GMP
*N*	17	18	19	23	16	22	18	16	14	19	20	13
tPY disp (mm)a,b	0.48±0.04	0.51±0.07	0.55±0.08	0.38±0.04	0.45±0.04	0.40±0.03	0.47±0.05	0.43±0.06	0.46±0.07	0.38±0.05	0.31±0.03	0.30±0.04
tTotal disp (mm)a,b	0.69±0.04	0.74±0.07	0.78±0.07	0.57±0.04	0.65±0.04	0.61±0.04	0.69±0.05	0.65±0.06	0.70±0.06	0.61±0.05	0.51±0.03	0.55±0.04
* rStiffness (N/mm)a,b,d,e	87±4	80±2	78±4	110±5	94±5	102±3	78±4	74±4	79±7	75±2	89±3	81±3
* rYield load (N)a,b	13.7±0.6	13.5±0.3	12.9±0.4	15.2±0.5	14.8±0.4	15.2±0.4	11.7±0.4	12.3±0.4	12.6±0.4	13.0±0.5	13.2±0.4	14.3±0.5
* rMax load (N)a,b,c,d	15.8±0.5	15.0±0.3	14.6±0.4	18.1±0.5	16.6±0.4	17.6±0.4	14.1±0.4	14.1±0.4	14.6±0.6	14.8±0.4	15.3±0.3	16.2±0.4
* tEnergy (N-mm)^a^	7.54±0.37	7.01±0.55	7.15±0.61	6.87±0.44	7.09±0.39	7.33±0.46	6.61±0.50	5.86±0.50	6.56±0.65	5.90±0.53	5.31±0.30	5.71±0.37

Values are means ± SE of raw data; *N*, no of mice; PY, post-yield; disp, displacement; N, newtons.

* Data analyzed using ANCOVA with a covariate of body mass.

t Data transformed to satisfy assumptions of normality and variance.

r Non-transformable data was ranked.

a genotype effect,

b sex effect,

c diet effect,

d gt*sex effect,

e gt*sex*diet effect.

**PY disp:** Males had a greater PY disp than females. WT mice had a greater PY disp than PKU mice.

**Total disp:** Males had a greater total disp than females. WT mice had a greater total disp than PKU mice.

**Stiffness:** All female groups except PKU females fed GMP or casein had stiffer bones than males. Among female groups WT females fed casein and GMP had the stiffest bones. There was no difference between female mice fed AA diet and female PKU mice fed GMP, however PKU females fed casein had a significant reduction in stiffness. Among male mice the only significant difference was a reduction in stiffness in male PKU mice fed AA compared to WT males fed casein.

**Yield load:** Female mice tolerated a higher load at the yield point than male mice. WT mice tolerated a higher load at the yield point compared to PKU mice.

**Max Load:** Females had a greater maximum load before fracture compared to males. Moreover, WT females had a greater max load than PKU females, however there were no difference between genotypes for the males. Mice fed the GMP diet had a significant increase in maximum load compared to mice fed the AA diet, but there was no difference between mice fed the GMP and casein diets, additionally there was no difference between mice fed the casein and AA diets.

**Energy:** It required more energy to fracture the femurs of WT mice than it did to fracture the femurs of PKU mice.

**Table 3:** Size, shape, and content of mice femora.

	Wild Type Mice						PKU Mice					
	Males			Females			Males			Females		
Variable	Casein	AA	GMP	Casein	AA	GMP	Casein	AA	GMP	Casein	AA	GMP
*N*	17	18	19	23	16	22	18	16	14	19	20	13
* tCSA (mm^2^)b,c,d	1.07±0.04	0.97±0.03	0.97±0.02	1.01±0.02	0.98±0.04	0.96±0.02	0.96±0.02	0.95±0.03	1.00±0.04	0.90±0.02	0.91±0.02	0.94±0.02
* rPerimeter (mm)b,c	6.07±0.12	5.78±0.12	5.80±0.10	5.60±0.05	5.47±0.08	5.37±0.04	5.80±0.07	5.60±0.09	5.72±0.12	5.49±0.07	5.34±0.06	5.38±0.07
* rInner Minor Axis (mm)a,b,c,e	0.93±0.03	0.90±0.02	0.91±0.02	0.89±0.01	0.85±0.01	0.84±0.01	0.92±0.01	0.88±0.02	0.87±0.01	0.93±0.01	0.86±0.01	0.88±0.02
* tInner Major Axis (mm)c,e	1.68±0.05	1.60±0.05	1.64±0.04	1.48±0.02	1.46±0.02	1.41±0.02	1.62±0.03	1.54±0.03	1.49±0.04	1.49±0.03	1.41±0.02	1.41±0.03
* rOuter Minor Axis (mm)b,c,e	1.34±0.03	1.27±0.02	1.29±0.02	1.32±0.01	1.27±0.02	1.27±0.01	1.29±0.01	1.25±0.02	1.27±0.02	1.31±0.01	1.24±0.01	1.28±0.02
* tOuter Major Axis (mm)^c^	2.25±0.05	2.11±0.05	2.11±0.03	2.02±0.02	1.97±0.03	1.93±0.02	2.11±0.03	2.06±0.04	2.10±0.05	1.95±0.03	1.90±0.03	1.92±0.02
*Shape Factor (unitless)^b^	1.68±0.02	1.66±0.03	1.64±0.02	1.54±0.02	1.55±0.02	1.52±0.01	1.64±0.02	1.65±0.03	1.65±0.02	1.49±0.01	1.53±0.02	1.50±0.02
* tCSMI (mm^4^)b,c,e	0.19±0.01	0.16±0.01	0.17±0.01	0.17±0.01	0.16±0.01	0.15±0.00	0.16±0.01	0.15±0.01	0.16±0.01	0.16±0.01	0.14±0.00	0.15±0.01
tBMD (mg/cm^2^)a,b,d	54.0±0.7	51.9±0.6	51.8±0.7	55.0±0.8	54.1±0.9	55.0±0.5	50.8±0.6	50.5±0.9	51.1±0.8	49.8±0.7	51.2±0.4	51.3±0.7
BMC (mg)a,b	29.2±0.6	27.3±0.5	27.0±0.6	27.8±0.6	27.1±0.5	27.5±0.4	26.0±0.5	25.5±0.8	25.6±0.7	23.3±0.6	24.6±0.3	24.4±0.7

Values are means ± SE of raw data; *N*, no of mice; CSA, cross sectional area; CSMI, cross sectional moment of inertia; BMD, areal bone mineral density; BMC, bone mineral content.

* Data analyzed using ANCOVA with a covariate of body mass.

t Data transformed to satisfy assumptions of normality and variance.

r Non-transformable data was ranked.

a genotype effect,

b sex effect,

c diet effect,

d gt*sex effect,

e gt*diet effect.

**CSA:** WT females had a greater CSA than PKU females, both of which were greater than the males per unit body weight. A main effect of diet demonstrated a significant reduction in CSA in mice fed the AA diet.

**Perimeter:** Females had a larger perimeter than males. Mice fed casein had the greatest perimeter, followed by GMP, and then AA.

**Inner minor axis (IMA):** Females had a larger IMA than males. PKU mice fed casein had the largest IMA. There was no difference between WT mice fed casein and WT or PKU mice fed GMP. Mice fed GMP were also not different from mice fed AA, however PKU mice fed AA had a significantly smaller IMA compared to WT mice fed casein.

**Inner Major Axis** **(IMAJA):** Mice fed the casein diet had the greatest IMAJA.

**Outer minor axis (OMA):** Females had a greater OMA than males. PKU mice fed casein had the longest OMA and PKU mice fed AA had the shortest.

**Outer major axis (OMAJA):** Mice fed casein had a larger OMAJA than mice fed either GMP or AA diet.

**Shape factor (SF)  =  OMAJA/OMA:** Females had a more circular cross section than males.

**CSMI:** Females had a larger CSMI compared to males after correction for body weight. PKU mice fed casein had the greatest CSMI. There was no difference in CSMI between WT mice fed casein, WT and PKU mice fed GMP and WT mice fed AA, however there was a significant reduction in CSMI in PKU mice fed AA. There also was no difference between WT and PKU mice fed casein or GMP.

**BMD:** WT females had a higher BMD than WT males, both of which were higher than the BMD of PKU mice, where there were no differences due to gender or diet.

**BMC:** Male mice had a greater BMC than females. WT mice had a greater BMC than PKU mice.

**Table 4:** Stress-strain curve analysis showing tissue level biomechanical performance.

	Wild Type Mice						PKU Mice					
	Males			Females			Males			Females		
Variable	Casein	AA	GMP	Casein	AA	GMP	Casein	AA	GMP	Casein	AA	GMP
*N*	17	18	19	23	16	22	18	16	14	19	20	13
* tYield strain (unitless)c,e	0.030±0.002	0.031±0.002	0.031±0.002	0.028±0.001	0.028±0.001	0.028±0.001	0.031±0.002	0.029±0.002	0.032±0.002	0.031±0.001	0.026±0.001	0.034±0.003
tPY strain (unitless)a,b	0.068±0.005	0.070±0.011	0.076±0.012	0.053±0.005	0.061±0.005	0.054±0.005	0.064±0.007	0.058±0.008	0.061±0.009	0.054±0.008	0.041±0.004	0.041±0.006
* tTotal strain (unitless)^e^	0.098±0.006	0.101±0.011	0.107±0.011	0.080±0.006	0.089±0.006	0.083±0.005	0.095±0.007	0.087±0.008	0.093±0.008	0.085±0.008	0.067±0.003	0.075±0.005
*Modulus (MPa)a,c,d,e,g	4184±256	4543±234	4305±231	5670±202	5374±223	6017±184	4236±253	4386±173	4520±274	4314±145	5768±234	4915±281
* rYield stress (MPa)a,b,c	92±3	102±4	96±3	111±3	115±4	121±3	86±2	98±2	98±3	103±3	112±4	117±4
* tMax stress (MPa)a,b,c,e,f	105±3	113±4	109±3	131±3	129±4	141±3	104±2	112±2	113±3	118±3	130±3	133±4
tToughness (MPa)^a^	7.14±0.29	6.97±0.40	7.19±0.51	6.95±0.43	7.51±0.47	7.87±0.45	6.74±0.50	6.13±0.45	6.84±0.63	6.39±0.49	5.95±0.31	6.39±0.45
* tFailure stress (MPa)^e^	80±4	76±7	72±6	99±5	91±5	105±3	76±5	84±5	82±4	92±5	104±4	106±5

Values are means ± SE of raw data; *N*, no of mice; PY, post-yield; MPa, megapascals.

* Data analyzed using ANCOVA with a covariate of body mass.

t Data transformed to satisfy assumptions of normality and variance.

r Non-transformable data was ranked.

a genotype effect,

b sex effect,

c diet effect,

d gt*sex effect,

e gt*diet effect,

f sex*diet effect,

g gt*sex*diet effect.

**Yield strain:** PKU mice fed GMP and casein had the highest strain at their yield point and PKU mice fed AA had the lowest. There was no difference between WT mice fed the three diets and PKU mice fed AA. Additionally, there was no difference between WT mice fed GMP and AA with PKU mice fed casein.

**PY strain:** Males had a greater strain after yielding than females. WT mice had a greater strain after yielding than PKU mice.

**Total strain:** PKU mice fed AA had a significant reduction in total strain and there was no difference across all other groups.

**Modulus:** The three way interaction demonstrated that females tended to have bones with a higher intrinsic stiffness than males; WT females fed GMP and PKU females fed AA had a higher modulus than WT and PKU males fed GMP as well as PKU males fed casein. Additionally, WT females fed casein or AA had a higher modulus than PKU males fed casein. Among females, PKU mice fed casein had a significant reduction in intrinsic stiffness, moreover WT females fed all three diets and PKU females fed AA had a higher modulus than PKU females fed GMP. Among males, PKU mice fed casein had a significant reduction in modulus compared to male mice fed AA and there was no difference across the other five groups.

**Yield stress:** Wild type mice tolerated more stress before yielding compared to PKU mice. Female mice tolerated more stress before yielding compared to male mice. Mice fed AA and GMP tolerated more stress before yielding compared to mice fed casein.

**Max stress:** Female groups fed GMP and AA had a higher max stress before fracture compared to male groups and female mice fed casein had a higher max stress than males fed casein. Among females, groups fed GMP had the highest max stress, followed by AA and then casein. Among males, groups fed AA had a higher max stress than males fed casein, but males fed GMP were not different from the other two. PKU mice fed casein had a significant reduction in max stress compared to WT and PKU mice fed GMP and AA as well as WT mice fed casein.

**Toughness:** WT mice had tougher bones than PKU mice, as measured by area under the stress strain curve.

**Failure stress:** PKU mice fed AA had a higher stress at fracture compared to WT mice fed AA and PKU mice fed casein. WT mice fed casein and PKU mice fed GMP also had a higher stress at fracture compared to PKU mice fed casein.

### Biomechanical Testing

We tested femoral diaphysis biomechanical performance by quasi-static 3-point bending under displacement control at a rate of 0.3 mm/sec, with a support span of 7.5 mm as previously described, **Figure 1B**. [27]. This produces a mid-diaphyseal fracture directly below the crosshead. By using the femoral condyles and the 3^rd^ trochanter as anatomical landmarks to position bones consistently, the testing protocol produces highly reproducible fractures. We obtained the periosteal perimeter, cortical cross-sectional area, outer and inner major and minor axis lengths, shape factor (ratio of outer major axis length to outer minor axis length), and cross-sectional moment of inertia in the fracture plane from digital photographs [27]. We used the geometric properties and the whole bone mechanical testing data to calculate the material properties of the bone tissue according to the standard beam theory equations [23], using the averages of both femora for further analysis:

Stress (σ), (MPa)  =  FLc/4*I* with F  =  force, L  =  length, c  =  outer radius in the plane of bending, and *I*  =  cross-sectional moment of inertia in the plane of bending.

Strain (ε), (mm/mm)  = 12cd/L^2^ with c  =  outer radius in the plane of bending, d  =  displacement, L  =  length.

Young’s Modulus (E), (MPa)  =  (F/d)(L^3^/48*I*) with F  =  force, L  =  length, and *I*  =  cross sectional moment of inertia in the plane of bending.

**Figure 3:**
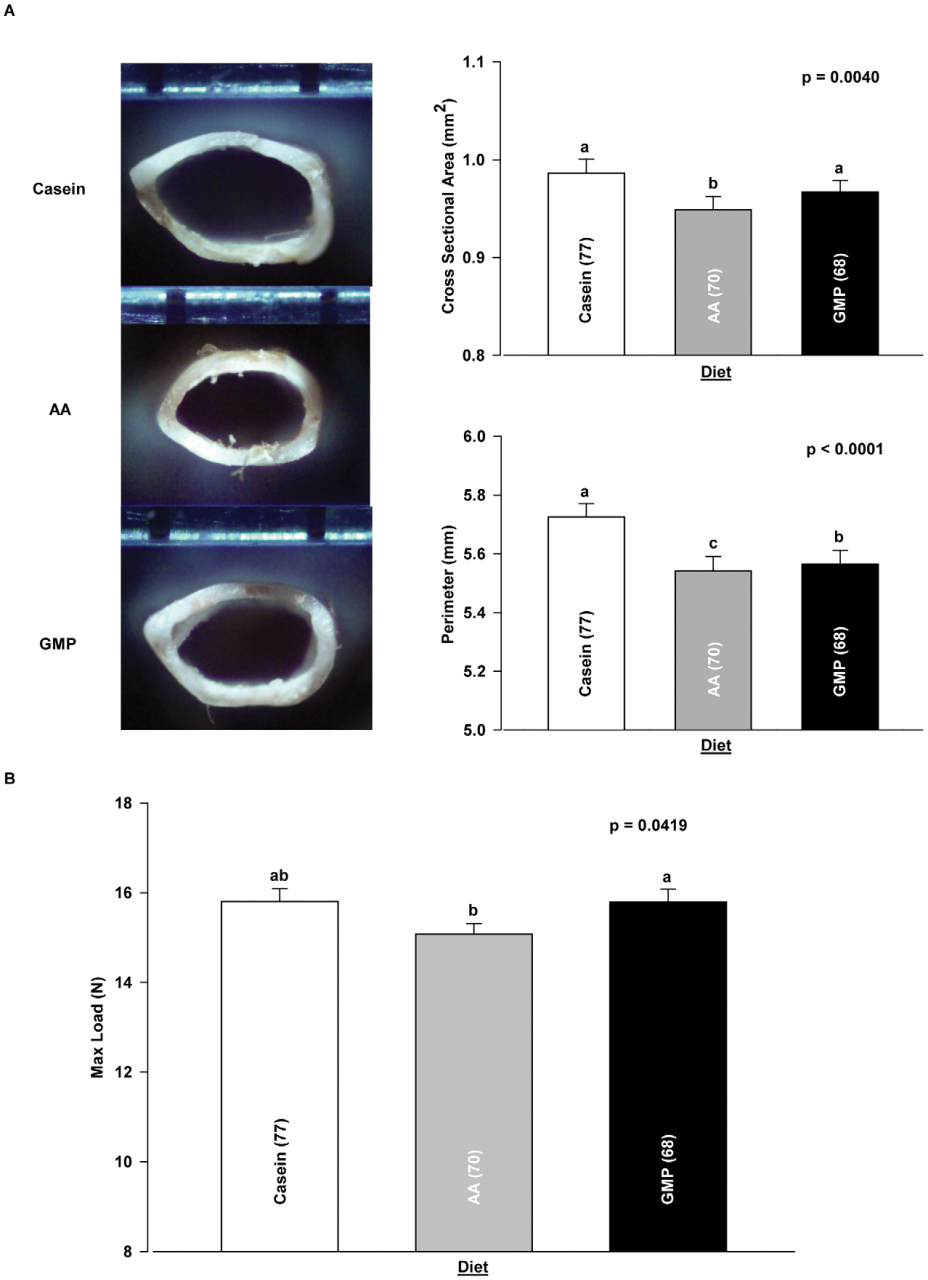
Diet modifies femoral size and strength in WT and PKU mice. Representative photographs of femoral cross-sectional geometry in mice fed casein, AA, and GMP diets from which measurements of cross-sectional area and perimeter are obtained (A). Diet effect on maximum load derived from load-displacement curve analysis (B). Values are means ± SE; p-values represent main effect of diet. Sample size is shown in parenthesis. Groups with different letter superscripts are significantly different (p<0.05).

### Principal Component Analysis

The following input variables were included in principal component analysis (PCA): Body mass, femoral BMD, post-yield deflection, total deflection, yield load, maximum load, energy, stiffness, femoral cross-sectional area, femoral periosteal perimeter, femoral inner major and minor axis lengths, femoral outer major and minor axis lengths, femoral diaphyseal shape factor, and femoral diaphyseal cross-sectional moment of inertia. The PCA was performed with the SAS function “proc princomp” (SAS Institute, Cary, NC) [28], [29], [30]. We performed further analysis of the principal components (PCs) with Eigenvalues ≥1. PC values for each animal were calculated by multiplying each PC’s Eigenvector by the animal’s parameter vector.

**Table 5:** Correlations among phenotypes.

	Mass	CSA	Perim	InMin	InMaj	OutMin	OutMaj	SF	CSMI	PYD	TD	Stiff	YLoad	MLoad	Energy	fBMD
Mass	–	0.45	0.63	0.30	0.56	0.29	0.67	0.61	0.47	0.27	0.29	−0.15	−0.08	−0.10	0.22	0.14
CSA		–	0.73	0.23	0.33	0.65	0.71	0.36	0.85	0.23	0.24	0.39	0.44	0.51	0.40	0.64
Perim			–	0.67	0.80	0.74	0.97	0.61	0.87	0.30	0.33	−0.01	0.11	0.10	0.29	0.31
InMin				–	0.70	0.78	0.58	0.09	0.65	0.10	0.10	−0.07	−0.01	0.01	0.03	0.06
InMaj					–	0.53	0.82	0.60	0.64	0.34	0.36	−0.16	−0.08	−0.12	0.22	0.11
OutMin						–	0.63	−0.04	0.91	0.13	0.14	0.25	0.33	0.40	0.22	0.42
OutMaj							–	0.75	0.79	0.31	0.35	−0.02	0.08	0.06	0.30	0.31
SF								–	0.25	0.29	0.33	−0.22	−0.17	−0.25	0.19	0.05
CSMI									–	0.26	0.27	0.28	0.35	0.42	0.36	0.53
PYD										–	0.98	0.00	−0.05	−0.02	0.86	0.11
TD											–	−0.10	−0.02	−0.05	0.84	0.09
Stiff												–	0.46	0.73	0.20	0.68
YLoad													–	0.85	0.30	0.67
MLoad														–	0.36	0.76
Energy															–	0.39
fBMD																–

Phenotypes are Mass, body mass; CSA, cortical cross-sectional area; Perim, periosteal perimeter; InMin, inner minor axis; InMaj, inner major axis; OutMin, outer minor axis; OutMaj, outer major axis; SF, shape factor; CSMI, cross-sectional moment of inertia; PYD, post-yield deflection; TD, total deflection; Stiff, stiffness; YLoad, yield load; MLoad, maximum load; Energy, energy to failure; fBMD, BMD by ex vivo DXA. Each cell shows R.

**Table 6:** Principal Component Analysis.

	PC1	PC2	PC3	PC4
Eigenvalue	6.68	3.57	2.39	1.29
Difference	3.11	1.19	1.09	0.72
r^2^	0.418	0.223	0.149	0.081
Cumulative r^2^	0.418	0.641	0.79	0.871

Difference is subtraction of subsequent PC from former PC.

### Statistical Analysis

Data were analyzed by three-way ANOVA or ANCOVA using “proc mixed”. Femoral biomechanical testing was performed on two different days due to the large number of femora, thus a random effect of time as a blocking factor was included in the model. The three-way ANCOVA tested for main effects of genotype, sex, and diet as well as their two and three way interactions. Femoral cross section measurements and biomechanical data were adjusted for the animal’s body mass by including body mass as a covariate. When body mass wasn’t a significant predictor for a parameter the term was removed and results from a subsequent three-way ANOVA are presented. Data presented are raw data or actual measurements, and the statistical significance shown represents the analysis adjusted for body weight based on ANCOVA where appropriate. Differences between treatment groups were detected using a protected Fisher's Least Significant Difference (LSD) test (SAS Institute, 2007, Cary, NC). Data transformations were performed where appropriate to fit assumptions of normality and equal variance prior to statistical analysis. If data transformations failed, a respective non-parametric ANCOVA or ANOVA was performed on ranked data. Untransformed data are presented in the tables. Data are analyzed per animal; biomechanical data are an average of the right and left femora. Data are presented as mean ± SE. P-values <0.05 are considered significant. Where there was no significant interaction, data were pooled into treatment groups by their respective significant main effects.

**Table 7:** Eigenvectors of Principal Components 1–4.

	PC1	PC2	PC3	PC4
Body mass	0.239	−0.205	−0.026	0.318
Cross-sectional area	0.316	0.161	−0.025	0.227
Perimeter	0.358	−0.125	−0.147	0.040
Inner minor axis	0.234	−0.111	−0.296	−0.474
Inner major axis	0.289	−0.242	−0.116	−0.077
Outer minor axis	0.302	0.107	−0.230	−0.375
Outer major axis	0.350	−0.153	−0.112	0.171
Shape factor	0.194	−0.282	0.053	0.538
CSMI	0.358	0.081	−0.143	−0.122
Post-yield deflection	0.183	−0.125	0.518	−0.203
Total deflection	0.189	−0.147	0.507	−0.186
Stiffness	0.083	0.409	0.036	0.074
Yield load	0.123	0.407	0.028	0.071
Max load	0.136	0.468	0.035	0.027
Energy	0.209	0.063	0.506	−0.116
Femoral BMD	0.209	0.362	0.051	0.202

CSMI, cross-sectional moment of inertia; BMD, bone mineral density.

**Table 8:** Principal component phenotypes of WT and PKU mice fed casein, AA, and GMP diets.

	Wild Type Mice						PKU Mice					
	Males			Females			Males			Females		
Variable	Casein	AA	GMP	Casein	AA	GMP	Casein	AA	GMP	Casein	AA	GMP
*N*	17	18	19	23	16	22	18	16	14	19	20	13
tPC1a,c,d	25.1±0.6	23.4±0.4	23.1±0.6	24.8±0.6	23.2±0.5	23.9±0.4	22.2±0.5	22.4±0.6	22.5±0.8	20.7±0.4	21.8±0.3	21.4±0.4
tPC2a,b,d,e	40.8±1.7	38.2±1.1	37.0±2.0	53.7±2.4	46.3±2.1	50.5±1.5	36.4±1.9	34.3±1.7	37.4±3.1	37.2±1.1	43.1±1.1	40.9±1.4
tPC3a,b	5.75±0.23	5.41±0.36	5.42±0.47	6.58±0.35	6.20±0.20	6.65±0.30	5.04±0.36	4.47±0.38	5.12±0.53	4.75±0.34	4.94±0.23	4.93±0.25
tPC4a,b,d	17.3±0.4	15.9±0.2	15.6±0.4	16.4±0.4	15.1±0.4	15.5±0.3	14.9±0.4	15.8±0.5	15.3±0.6	13.3±0.3	14.7±0.2	14.0±0.4

Values are means ± SE; *N*, no of mice; PC, principal component.

t Data transformed to satisfy assumptions of normality and variance.

a genotype effect,

b sex effect,

c gt*sex,

d gt*diet effect,

e gt*sex*diet effect.

**PC1:** WT mice fed Casein had a significant increase in their calculated PC1 values compared to WT mice fed GMP or AA. PKU mice on the three diets had significantly lower values than WT mice. Additionally, PKU males and females had lower values than WT males and females of which PKU female values were the lowest.

**PC2:** WT males fed GMP had reduced values compared to WT males fed casein, but were not different from WT males fed AA. There were no differences within the PKU male groups on the three diets, however PKU males fed AA had lower values than WT males fed AA or casein. There were no differences between the values within the WT females groups, however WT females fed AA were not different from PKU females fed GMP or AA, whereas WT females fed GMP or casein were significantly higher. PKU females fed casein had a significant reduction compared to PKU females fed AA, but not GMP. Additionally, WT females had higher values than WT males, and PKU females had higher values than PKU males with the exception of PKU females fed casein.

**PC3:** WT mice had greater values than PKU mice, and females had greater values than males.

**PC4:** WT mice fed casein had greater values than WT mice fed GMP or AA. PKU mice fed the three diets had a significant reduction in values compared to WT mice; however PKU mice fed AA were not different from WT mice fed GMP or AA. Male mice had greater values than female mice.

## Results

### Growth and Whole Body Bone Mineral Density

The low-phe AA and GMP diets significantly reduced plasma phe concentration in both WT and PKU mice, **Table 1** **.** In spite of restricting dietary phe to the minimum needed to support nearly normal growth, plasma phe concentration in PKU mice remained abnormally elevated, approximately 14-fold higher in PKU compared with WT mice. Regardless of diet, whole body BMD was significantly lower in PKU compared with WT mice. Regardless of sex, femur length was significantly shorter in WT mice fed the AA diet compared with the GMP and casein diets, and not significantly different from PKU mice. Plasma phe concentration did not predict femur length, whole body BMD or BMC.

### Femora of PKU Mice Have Decreased Strength and Increased Brittleness

The 3-point bending test produces mid-diaphyseal fractures under controlled loading conditions (Figure 1B **)**. The primary analysis of the test measures 3 elements of biomechanical performance: displacement (the amount the bone bends), strength (load, or the force applied to the bone), and absorbed energy (the area under the load-displacement curve), as summarized in **Figure 2A** **and** **Table 2**. Representative load-displacement curves for a WT and PKU femur are shown in **Figure 2B**. Following fracture, specimen geometry in the plane of the fracture is assessed (**Table 3**), allowing calculation of the corresponding tissue-level mechanical properties (**Table 4** **)** from standard beam equations, as described in the methods section. The raw data reflect the biomechanical performance of the actual bone, while the tissue level or material properties (i.e., tissue properties and material properties are synonyms) reflect the performance of an idealized sample of the bone tissue, independent of its size and shape.

Regardless of sex or diet, the PKU genotype was associated with reduced femoral biomechanical properties assessed by three-point bending **Figure 2B–H**. Yield load, or the force required to elicit permanent damage to the femur, and the maximum load achieved before fracture were significantly lower in PKU mice. Total displacement, or the total amount of deformation by the femur before fracture, was also reduced in PKU compared with WT mice. The post-yield displacement, a measurement of ductility, was significantly lower with PKU, thus the femora of PKU mice are more brittle (i.e., the opposite of ductile) and yield sooner than WT femora. The stiffness of PKU bones was lower than WT bones. Additionally, the energy required to fracture the bone, measured as the area under the load-displacement curve, was significantly reduced in PKU femora. These measures of whole bone biomechanical performance are paralleled by the tissue level analysis of the stress-strain relationship (Table 4). This suggests an inherent disturbance in the biomechanical performance of PKU bone tissue, independent of size. Moreover, decreases in mineralization are supported by *ex vivo* DXA data. Both areal BMD as well as BMC of femora (Table 3) are significantly reduced in PKU mice compared with WT mice. In summary, the femora in PKU mice display global deficits of biomechanical performance, and are more brittle, weaker, less stiff, and absorb less energy than those of WT mice.

We sought evidence regarding whether plasma phe levels are related to bone status. We found no significant relationship between plasma phe and any biomechanical or BMD outcomes (data not shown).

### Dietary Protein Source Modifies the PKU Bone Phenotype

Regardless of genotype and sex, the AA diet reduced femoral size, as manifested in a significant reduction in femoral cross sectional area (CSA) and in the femoral perimeter, compared with the casein and GMP diets (**Figure 3A** **)**. Femoral size expressed as the cross sectional moment of inertia (CSMI), a measure of the distribution of material around a neutral axis, was also significantly lower in PKU mice fed the AA diet compared with the casein and GMP diets (Table 3). Consequent to the reduction in femoral size, both WT and PKU mice fed an AA diet tolerated a lower maximum load compared with the GMP diet, **Figure 3B**. Tissue level analysis across diet treatments provides supporting evidence that the AA diet yields a more brittle and weaker bone compared to the GMP diet in that it decreases yield strain, total strain, and maximum stress in PKU mice (**Table 4**). In summary, the reduction in both femoral size and maximum load in mice fed the low-phe AA diet suggests that providing dietary protein from GMP rather than AA attenuates the PKU bone phenotype.

### Principal Components Analysis

The bone properties we measured in this study are not mutually independent. For example, yield load and maximum load are highly correlated with an R of 0.85 (**Table 5**). Furthermore, with so many properties measured, one might ask which are most important. PCA provides a linear transformation of the raw data to an equal number of mutually orthogonal PCs, each of which is a linear function of the raw data [31], [32]. By established convention, further analysis is limited to those PCs with Eigenvalues exceeding 1. The goal of the analysis is to explain the experimental variation while reducing the dimensionality of the data and maintaining the statistical independence of the PCs. PCA of 16 whole bone femoral measurements yielded four PCs with Eigenvalues >1, which collectively accounted for 87% of the variance (**Table 6**).

The PCs are composites of all the measured parameters, but can be interpreted on the basis of the coefficients for each, as summarized in the Eigenvectors (**Table 7**). PC1 is “size-like,” with cross-sectional area, perimeter, inner major axis, outer minor axis, outer major axis, and cross-sectional moment of inertia contributing most to it. PC2 is “strength-like,” but at the material rather than the whole-bone level, with large contributions from stiffness, yield and maximum load, and BMD, but with negative contributions from multiple size parameters. PC3 is “ductility-like,” with post-yield deflection, total deflection, and energy to failure prominent contributors. PC4 is the most difficult to interpret, with body mass, cross-sectional area, shape factor, and areal BMD of femora having large coefficients. Regardless of their interpretation, it is notable that there are significant genotype-dependent differences in all 4 PCs, indicating that the bone phenotype in PKU mice represents a global deficit in biomechanical performance (**Table 8**).

## Discussion

Success in managing the neurological manifestations of PKU by dietary phe restriction has allowed many patients to enjoy a greatly improved prognosis, spared of devastating cognitive impairment. Because of their improved function, other, more subtle deficits have become apparent [16]. Chief among these is skeletal fragility, as noted by multiple independent research groups [3], [4], [5], [6], [7], [8], [9], [10], [11]. However, since dietary phe restriction is initiated within days of birth, it has previously not been possible to distinguish whether skeletal fragility is a manifestation of PKU itself, or of its dietary treatment. The availability of an animal model of PKU, harboring an ENU-induced point mutation of the murine *Pah* gene allowed this uncertainty to be resolved experimentally [21].

Our data show that PKU mice have impaired bone biomechanical performance, regardless of the effects of sex or diet. The biomechanical deficit is complex, encompassing strength, stiffness, and ductility. PCA reveals a genotype effect for all 4 PCs, confirming the principal findings of the whole bone biomechanics. Bone is a composite tissue composed of a protein matrix, containing primarily type 1 collagen, and precipitated mineral, containing primarily calcium and phosphate in the form of apatite [33]. The protein elements of bone matrix contribute tensile strength and ductility to the composite, while the mineral provides compressive strength and stiffness [34], [35]. These are distinct properties, both of which are needed to resist fracture. In our study, strength, stiffness, and ductility are reduced in PKU mice relative to WT mice. The finding that all 4 PCs of biomechanical performance differ significantly between genotypes further supports the inference that multiple aspects of bone function are impaired in the PKU mice relative to WT mice.

While our data clearly support the existence of deficits in bone strength and ductility in PKU mice, the present data are insufficient to identify the biochemical, cellular, and physiological mechanisms underlying them. The organic components of bone matrix are produced by osteoblasts and these are mineralized following secretion into the extracellular space [34], [36]. Matrix synthesis is a complex, hierarchically organized process, so that abnormalities at early stages will have cascading effects at higher levels of structure [35], [37]. Increased brittleness could arise as a consequence of a primary abnormality of type 1 collagen, its cross-linking, its assembly, abnormalities of other matrix proteins, disorganization of matrix assembly, or a combination of these [34], [38], [39]. Decreased strength reflects a quantitative deficit of mineralization, as suggested by our observation of decreased BMD in PKU mice, but changes in the composition or crystal structure could also contribute to reduced strength.

It is important to recall that bone is known to model in response to its usual loading environment, and bone size and strength therefore vary as a function of body size and activity [33], [40]. PKU mice are smaller than WT mice, resulting in lower loads if activity were equivalent in the 2 genotypes. We adjusted our data for body mass prior to comparison of biomechanical performance, so our results have accounted for differential growth. However, our experiment did not assess cage activity while the mice were living. It is possible that neurological damage arising from phe toxicity leads to reduced activity, and that the activity difference contributes to the observed differences in skeletal phenotypes. Assessing whether PKU bones respond similarly to WT bones to defined loading conditions *in vivo* remains an important question for future research.

Our data also demonstrate that dietary protein source consistently affects both size and strength in WT and PKU mice. The AA diet impairs the radial growth of the femur, affecting all diaphyseal dimensions relative to casein (Table 3). The GMP and casein diets yielded larger femoral dimensions than the AA-based diet. This occurs in both WT and PKU mice, and is important because biomechanical performance at the whole bone is highly dependent on radial size [41]. Insufficient amounts of AA that are abundant in type 1 collagen, such as glutamic acid, arginine, lysine and proline, is one possible reason for this. Type 1 collagen comprises approximately 90% of the protein in bone matrix, and during skeletal growth, it is synthesized and degraded at a rapid rate. Subtle deficiency of amino acids that are abundant in collagen might therefore be manifested by reduced skeletal modeling. This possibility is attractive because it can account for reduced bone growth in mice fed the AA diet. A second possibility, applicable only to the AA diet, is that the acid load imposes a metabolic burden that restricts skeletal modeling. We previously showed that PKU mice fed the AA diet experience metabolic stress as evidenced by an increase in food and water intake, and renal hypertrophy compared to mice fed a GMP diet [22]. Moreover, PKU mice fed the AA diet had an increase in energy expenditure not different from the elevation observed in PKU mice fed casein. The experiments reported here do not address whether these mechanisms contribute to restricted radial bone growth, but are readily testable.

The work reported here can only be compared to prior studies in limited ways. A prior experiment reported that 8 weeks of phe restriction improved bone status in PKU mice [42]. However, the technical quality of the bone characterization was inferior to our methods, and the work was conducted in a BTBR/J rather than a C57BL/6J background. The human literature includes several reports addressing the relationship between plasma phe and BMD. The data are equivocal on this point, with some showing a negative correlation [7], [9], [10] while other studies do not [3], [4], [6], [43]. A human cohort study reported that low BMD is apparent in patients with PKU from an early age and that the deficit remains stable over time [3]. However, since all subjects were prescribed low-phe AA diets, the contributions of genotype and diet could not be resolved. Human work has primarily been cross-sectional and the bone characterization has been limited to clinical fractures, BMD, and serum markers of bone turnover [3], [4], [5], [6], [7], [8], [9], [10], [43], [44], [45], [46]. Use of a mouse model allowed us to overcome these limitations. We recently reported an increase in systemic inflammation as evidenced by significant splenomegaly and increases in inflammatory cytokines in PKU mice fed AA or casein diets; this response was normalized with the GMP diet [22]. Our observations parallel findings in human PKU. Roato et al. reported that activated T-cells, a major source of tumor necrosis factor alpha, induced spontaneous osteoclastogenesis which was associated with decreased bone status in human PKU [47].

Our experiment featured several notable strengths. Use of a uniform C57BL/6J genetic background eliminated possible confounding due to the segregation of genes other than *Pah*, and sample sizes were adequate to power main effect comparisons of all important biomechanical endpoints [48]. The biomechanical characterization of the bones was comprehensive and used robust methods. Lastly, diets were isoenergetic allowing the animals’ growth and metabolism to be characterized in detail [22].

Several limitations of our work must also be acknowledged. The C57BL/6 background is inbred, and therefore not reflective of the varied, outbred genetic backgrounds encountered in human PKU. The 3 point bending test, while robust and reproducible, produces experimental fractures in the mid-diaphysis of the femur, a site that is not generally susceptible to clinical fracture. Moreover, this is a skeletal site that is composed of cortical bone, so our data do not address the consequences of PKU on trabecular bone. Mice, because of their small size, do not have the osteonal structure characteristic of human cortical bone. The diets were only started at 3 weeks of age, when mice are weaned. This is distinct from PKU management in humans, which features dietary restriction beginning in the first week following birth. However, since long bones in mice undergo extensive linear and radial growth between weaning and young adulthood, most, if not all the femoral diaphyseal bone present at the time of testing was synthesized during the course of study. Finally, while our study was adequately powered to detect genotype, diet, and sex differences in all aspects of biomechanical performance at the main effect level, the sample sizes were insufficient to detect the impact of interactions on energy, post-yield displacement, and their material level correlates.

In summary, the data reported here demonstrate that skeletal fragility is an intrinsic feature of PKU in mice. The biomechanical defects are complex, affecting both strength and ductility. In mice, an AA diet exacerbates skeletal fragility by limiting radial bone growth, which is attenuated by a GMP diet. As an AA diet is presently the standard of care, this finding suggests that there is a need to determine whether improved diets can improve bone health in patients with PKU. The mechanisms by which PKU causes bone fragility and the AA-based diet impairs radial bone growth remain unknown, illustrating the need for further work to fully define the skeletal pathophysiology of PKU and its treatment.
